# Examining the Capacity of Text Mining and Software Metrics in Vulnerability Prediction

**DOI:** 10.3390/e24050651

**Published:** 2022-05-05

**Authors:** Ilias Kalouptsoglou, Miltiadis Siavvas, Dionysios Kehagias, Alexandros Chatzigeorgiou, Apostolos Ampatzoglou

**Affiliations:** 1 Centre for Research and Technology Hellas, 57001 Thessaloniki, Greece; siavvasm@iti.gr (M.S.); diok@iti.gr (D.K.); 2 Department of Applied Informatics, University of Macedonia, 54636 Thessaloniki, Greece; achat@uom.edu.gr (A.C.); a.ampatzoglou@uom.edu.gr (A.A.)

**Keywords:** vulnerability prediction, dataset extension, software metrics, text mining, machine learning, deep learning, ensemble learning

## Abstract

Software security is a very important aspect for software development organizations who wish to provide high-quality and dependable software to their consumers. A crucial part of software security is the early detection of software vulnerabilities. Vulnerability prediction is a mechanism that facilitates the identification (and, in turn, the mitigation) of vulnerabilities early enough during the software development cycle. The scientific community has recently focused a lot of attention on developing Deep Learning models using text mining techniques for predicting the existence of vulnerabilities in software components. However, there are also studies that examine whether the utilization of statically extracted software metrics can lead to adequate Vulnerability Prediction Models. In this paper, both software metrics- and text mining-based Vulnerability Prediction Models are constructed and compared. A combination of software metrics and text tokens using deep-learning models is examined as well in order to investigate if a combined model can lead to more accurate vulnerability prediction. For the purposes of the present study, a vulnerability dataset containing vulnerabilities from real-world software products is utilized and extended. The results of our analysis indicate that text mining-based models outperform software metrics-based models with respect to their F_2_-score, whereas enriching the text mining-based models with software metrics was not found to provide any added value to their predictive performance.

## 1. Introduction

Modern software programs are typically large, complicated, and interconnected. To design secure software, it is vital to follow secure and good programming methods. As a result, strategies and approaches that can offer developers with indicative information on how secure their software is are needed to help them improve their security level. Vulnerability prediction techniques may provide reliable information regarding software’s vulnerable hotspots and assist developers in prioritizing testing and inspection efforts by assigning limited testing resources to potentially vulnerable areas. Vulnerability Prediction Models (VPMs) are often created using Machine Learning (ML) approaches that utilize software features as input to differentiate between vulnerable and clean (or neutral) software components. Several VPMs have been developed throughout the years, each of which uses a different set of software features as inputs to anticipate the presence of vulnerable components (e.g., software metrics [1,2,3], text features [4,5], static analysis alerts [6,7], etc.).

More specifically, the initial attempts in the field of software vulnerability prediction investigated the ability of software metrics to indicate vulnerability existence in software, paying more focus on cohesion, coupling, and complexity metrics [1,2,3]. They utilized ML algorithms to classify software components as vulnerable or not. Text mining approaches, where researchers tried to extract text patterns from the source code utilizing Deep Learning (DL) models, were also examined [4,5,8,9], and demonstrated promising results in vulnerability prediction. Although both approaches have been studied individually, and there are several claims that text mining-based approaches lead to better vulnerability prediction models, to the best of our knowledge, apart from [10,11], there is a lack of studies that directly compare text mining-based with software metrics-based vulnerability models or studies that examine the combination of text features and software metrics as indicators of vulnerability.

The aforementioned research challenges, which constitute the main focus of the present work, can be formally expressed in the following Research Questions (RQ):

- RQ-1: Are text mining-based models better in vulnerability prediction than those utilizing software metrics?
- RQ-2: Can the combination of text features and software metrics lead to more accurate vulnerability prediction models?

More specifically, in the present paper, we investigate whether using text mining-extracted features can lead to adequate vulnerability prediction performance and we compare the resulting models to software metrics-based models. We also investigate whether combining software metrics with text features could result in more accurate vulnerability prediction models. To achieve this, we utilize a vulnerability dataset provided by Ferenc et al. [12] containing vulnerabilities from real-world open-source software applications, and extend it by adding additional features extracted through text mining (e.g., BoW and token sequences). Then, we replicate the work provided by Ferenc et al. [12] in which the authors used the aforementioned dataset and ML models in order to predict vulnerable functions, based on software metrics. Subsequently, we build our own DL models based on text mining and compare their predictive performance with the software metrics-based models. Finally, we attempt to combine these two kinds of inputs and train an Ensemble learning classifier [13], in order to examine whether the combination of text features and software metrics can lead to more accurate vulnerability prediction models.

The rest of the paper is structured as follows. In Section 2, the necessary theoretical background is provided in order to familiarize the reader with the main concepts of the present work. In Section 3, the related work in the field of Vulnerability Prediction in software systems is presented. Section 4 provides information about the adopted methodology. Section 5 discusses the results of our analysis and Section 7 concludes the paper also providing a discussion of potential future research directions.

## 2. Theoretical Background

In this section, we present the theoretical background of vulnerability prediction in general and the specific technologies that we have used as part of the work that is described in the present paper. This information is critical for familiarizing the reader with the concepts of Vulnerability Prediction, both text mining-based and software metrics-based. The ensemble learning background is described as well.

### 2.1. Vulnerability Prediction

**Figure 1:**
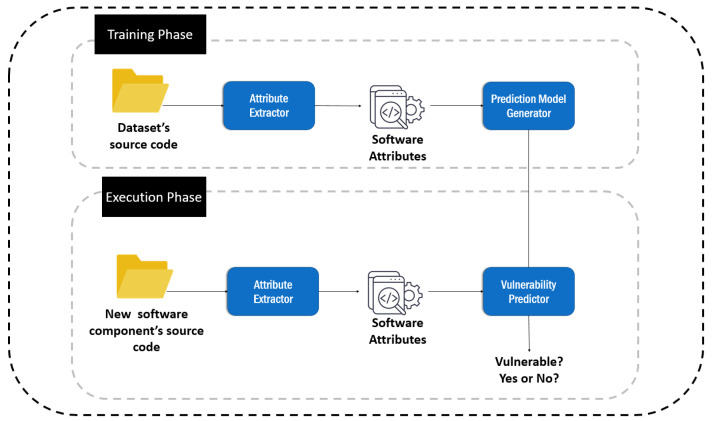
The basic concept of vulnerability prediction.

The purpose of Vulnerability Prediction is to identify software hotspots (i.e., software artefacts) that are more likely to contain software vulnerabilities. These hotspots are actually parts of the source code that require more attention by the software developers and engineers from a security viewpoint. Vulnerability Prediction Models (VPMs) are models able to detect software components that are likely to contain vulnerabilities. These models are normally built based on Machine Learning (ML) and are used in practice for prioritizing testing and inspection efforts, by allocating limited test resources to potentially vulnerable parts. For better understanding, the general structure of a Vulnerability Prediction Model is depicted in Figure 1.

As can be seen by Figure 1, the core element of vulnerability prediction is a vulnerability predictor, a model that is used to decide whether a given source code file (i.e., software component) is potentially vulnerable or not. The first step of the process is the construction of the vulnerability predictor. In order to construct the vulnerability predictor, a repository of clean and vulnerable software components (e.g., classes, functions, etc.) is initially constructed. Subsequently, appropriate mechanisms are employed in order to extract attributes from the source code (e.g., software metrics, static analysis alerts, text features, etc.), which are collected in order to construct the dataset that will be used for training and evaluating vulnerability prediction models. Then several VPMs are generated and the one demonstrating the best predictive performance is selected as the final vulnerability predictor. During the execution of the model in practice, when a new source code file arrives to the system, its attributes are extracted and provided as input to the vulnerability predictor, which, in turn, evaluates whether it is vulnerable or not.

The selection of the type of the attributes that will be provided as input to the generated VPMs is an important design decision in Vulnerability Prediction. The main VPMs that can be found in the literature are based on software attributes extracted from the source code either through static analysis (e.g., such as software metrics) [1,2,3] and text mining (e.g., bag of words, sequences of tokens, etc.) [4,5,9].

Software metrics-based VPMs: When the VPMs utilize software metrics, they are trained on numerical features that describe some characteristics of the source code (e.g., complexity, lines of code, etc.). These metrics are commonly extracted through static analysis and can provide quantitative information about quality attributes of the source code, such as the number of function calls and the number of linearly independent paths through a program’s source code. Popular metric suites that are used in practice are the Chidamber & Kemerer (CK) Metrics [14] and Quality Model for Object Oriented Design (QMOOD) [15] metric suites. Several open- and closed-source tools are available for their calculation, such as the (Chidamber & Kemerer Java Metrics) CKJM Extended (http://gromit.iiar.pwr.wroc.pl/p_inf/ckjm/, accessed on 2 January 2022), and the Understand (https://en.wikipedia.org/wiki/Understand_(software), accessed on 2 January 2022) tools.

Text mining-based VPMs: On the other hand, text mining-based VPMs are trained on datasets made up of text tokens retrieved from the source code. The simplest text mining approach is Bag of Words (BoW). The code in BoW is separated into text tokens, each of which has a count of how many times it appears in the source code. As a result, each word represents a feature, and the frequency of that feature in a component equals the feature’s value in that component. Apart from BoW, a more complex text mining approach involves the transformation of the source code into a list of token sequences that can be fed into Deep Learning (DL) models that can parse sequential data (e.g., recurrent neural networks). The token sequences are the input to the DL models, which try to capture the syntactic information in the source code during the training phase and anticipate the presence of vulnerabilities in software components during the execution phase. To extract semantic information from tokens, text mining-based methods also employ Natural Language Processing (NLP) techniques including token encoding with word2vec (https://radimrehurek.com/gensim/models/word2vec.html, accessed on 10 December 2021) embedding vectors. Word embedding methods learn a real-valued vector representation for a predetermined fixed-sized vocabulary from a corpus of text [16]. On a given natural language processing task, such as document classification, an embedding layer is a word embedding trained in combination with a neural network. It needs cleaning and preparing the document text in order for each word to be encoded in a one-hot vector. The size of the vector space is determined by the model. Small random numbers are used to seed the vectors. The embedding layer is utilized at the front end of a neural network and is fitted using the Backpropagation method in a supervised way.

### 2.2. Ensemble Learning

The ensemble learning [13] is a machine learning meta method that aims to improve predictive performance by integrating predictions from various models. It is actually an ML technique that combines numerous base models to build a single best-predicting model. The core premise of ensemble learning is that by merging many models, the faults of a single model will most likely be compensated by other models, resulting in the ensemble’s total prediction performance being better than that of a single model. The most common ensemble methods are divided into three categories, namely bagging, boosting, and stacking.

Bagging [17,18] is a technique used to reduce prediction variance by fitting each base classifier on a random subset of the original dataset and subsequently combining their individual predictions (either by voting or average) to generate a final prediction. Boosting [18] is an ensemble modeling strategy that aims to create a strong classifier out of a large number of weak ones. It is accomplished by constructing a model from a sequence of weak models. To begin, a model is created using the training data. The second model is then created, which attempts to correct the faults in the first model. This approach is repeated until either the entire training data set is properly predicted or the maximum number of models has been added.

**Figure 2:**
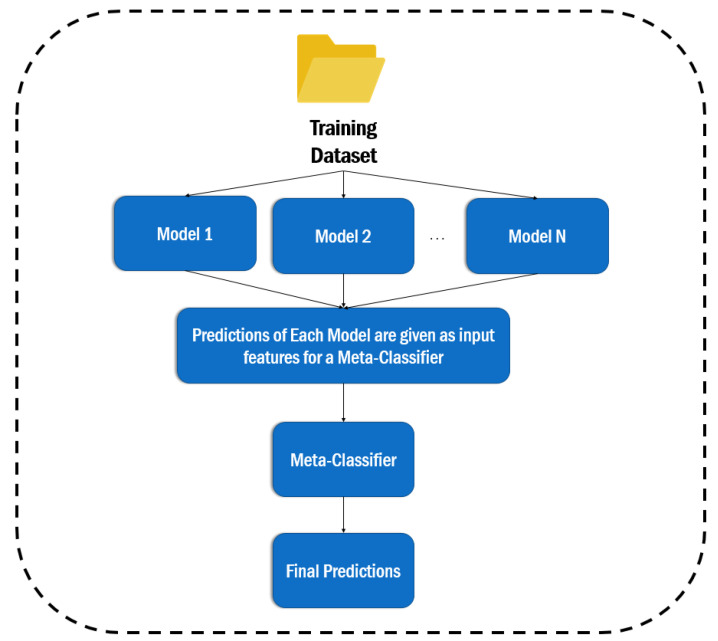
The architecture of the Stacking classifier.

In this study, the stacking classifier is employed (see Section 4.3). Stacking (https://towardsdatascience.com/stacking-classifiers-for-higher-predictive-performance-566f963e4840, accessed on 2 January 2022) is a technique for bringing together models. It is made up of two-layer estimators. The baseline models that are used to forecast the outcomes on the validation datasets make up the first layer, while the meta-classifier constitutes the second layer, which takes all of the baseline model predictions as input and generates new predictions, as can be seen in the Figure 2.

## 3. Related Work

Vulnerability prediction is a relatively new research topic in software security that seeks to predict which software components are likely to have vulnerabilities. Its goal is to find algorithms that can be used as indicators of software security vulnerabilities, identifying components as either potentially vulnerable or neutral. Vulnerability prediction models (VPMs) are created for this purpose using machine learning techniques and software properties as input. Using static analysis metrics [1,2,3] and/or text mining [4,5] are widespread techniques to build VPMs.

Software metrics-based Vulnerability Prediction: Shin and Williams [1,2] were the first researchers to look into the capacity of software metrics, particularly complexity metrics, to predict vulnerabilities in software products. To distinguish vulnerable from non-vulnerable functions, several regression models were created. According to their findings (which were based on the Mozilla JavaScript Engine), complexity measurements are only good indicators of software vulnerabilities. Chowdhury and Zulkernine [3] proposed a paradigm for predicting vulnerabilities based on CCC metrics (complexity, coupling, and cohesion). They compared the predictions of four distinct algorithms—Decision Tree, Random Forest (RF), Logistic Regression, and Naive-Bayes—using 52 versions of Mozilla Firefox. They came to the conclusion that structural data from non-security domains such as CCC is valuable in vulnerability prediction.

Kalouptsoglou et al. evaluated if combining artificial neural networks with software measurements could lead to more accurate cross-project vulnerability prediction [19]. On the basis of a dataset of well-known PHP products, several machine learning (including deep learning) models were built, assessed, and compared. Aiming to see if feature selection has an effect on cross-project prediction, feature selection is also used. They noticed that models that were constructed based on a certain set of software projects seem to deliver superior results when applied to new software projects that demonstrate similarities with respect to the significance of their features to the occurrence of vulnerabilities. Moshtari et al. [20] investigated the potential of software complexity to predict vulnerabilities across several software projects (i.e., cross-project prediction). They also compared the predictive value of complexity and coupling in cross-project prediction [21]. The results showed that complexity metrics had better discriminative ability in cross-project prediction than coupling metrics, and that combining traditional complexity measurements with a newly proposed set of coupling metrics improved the recall of the best complexity-based VPM built in this study.

Text mining-based Vulnerability Prediction: In text mining approaches, the source code of software components is parsed and represented as a set of code-tokens, which are then used to train predictors. Vulture [8], a VPM that predicted vulnerabilities based on import statements and function calls that are more common in vulnerable components, was the first framework to be suggested. Vulture was tested on Mozilla Firefox and Thunderbird code, and the findings were positive. Hovsepyan et al. [9] proposed a more comprehensive text mining-based prediction technique. They parsed the source code of software components to extract text items and their frequencies, which they used as predictive features (i.e., Bag of Words). An empirical study of their technique on 19 versions of a large-scale Android application found that it could be useful for vulnerability prediction, since the derived predictors had appropriate precision and recall.

Instead of employing raw text features, Pang et al. [4] used N-Gram analysis (https://towardsdatascience.com/understanding-word-n-grams-and-n-gram-probability-in-natural-language-processing-9d9eef0fa058, accessed on 1 January 2022) to describe source code as continuous token sequences. They used a deep neural network to identify vulnerable software components and integrated N-gram analysis and statistical feature selection for building features, evaluating their findings on a number of Java Android programs. The results of the evaluation demonstrated that the approach can deliver high precision, accuracy, and recall ideas with high precision, accuracy, and recall. However, because the evaluation was based on a small dataset, additional analysis would be required to determine that the findings were generalizable. Li et al. introduced a deep learning model for vulnerability detection in their paper VulDeePecker [5]. They divided the original code into a number of semantically linked lines of code, which they subsequently converted into vectors using the word2vec program. They developed a Bidirectional LSTM (BLSTM) model to detect library/API function calls linked to known flaws.

Vulnerability Prediction using both software metrics and text features: In terms of combining software metrics and text mining, no advanced models have been provided in the literature that can integrate text tokens with knowledge acquired from software metrics. Zhang et al. proposed VULPREDICTOR [11], an approach that investigates whether a combination of text and software metrics could lead to superior results. The evaluation results suggest that the combination of software metrics with text mining may be promising for vulnerability prediction, as they outperformed the results produced by Walden et al. [10], who used software metrics or text mining separately. In [22], the authors proposed an approach called HARMLESS, which employs a semi-supervised model to predict the remaining vulnerabilities in a code base using a Support Vector Machine (SVM) prediction model with undersampled training data. HARMLESS identifies which source code files are most likely to have flaws. In their case study, they also used Mozilla’s code base, with three different feature sets; metrics, text, and a combination of text mining and crash features, which actually describe the number of times the source code file has crashed.

Open Issues and Potential Contributions: As regards the comparison between text mining and software metrics as indicators for vulnerability existence, a limited number of attempts can be found in the literature. Walden et al. [10] compared text mining-based vulnerability prediction models to models that used software metrics as predictors. Their analysis was based on a dataset including 223 vulnerabilities discovered in three web applications for this purpose (i.e., Drupal, Moodle, and PHPMyAdmin). Random Forest models were trained to predict vulnerable and clean PHP files in their study. The findings revealed that text mining outperforms software metrics when it comes to project-specific vulnerability prediction, but it falls short in cross-project vulnerability prediction, where software metrics perform better. The results of this analysis do not clearly indicate which approach is superior and also it is based on a limited number of vulnerabilities and programming languages. Furthermore, to the best of our knowledge, apart from VULPREDICTOR [11] and HARMLESS [22], there are no other studies examining the benefits of combining software metrics and text features. There is a need for further research in this direction in order to enhance the generalizability of the outcomes of these studies.

In the present work, we attempt to address these open issues through an empirical analysis. In particular, we utilize text mining in order to build vulnerability prediction models and examine whether they indeed lead to highly accurate predictive performance using a real-world dataset constructed by Ferenc et al. [12], which we extended appropriately for the purposes of the present work. For the construction of the text mining-based models we utilize popular word embedding vector algorithms, namely word2vec and fastText, along with Deep Learning algorithms. Apart from text mining, we also investigate whether the utilization of software metrics could lead to sufficient vulnerability prediction performance, and we compare the produced models with text mining-based models. Finally, we examine whether the combination of software metrics with text features could lead to more accurate unified vulnerability prediction models, either by jointly building models that combine both types of features or by combining the outputs of independent software metrics-based and text mining-based models through a meta-classifier based on the voting and stacking ML paradigms.

## 4. Materials and Methods

In this section, the overall methodology that we adopted for building (i) the individual text mining-based and software metric-based models, and (ii) the combinatorial model that considers both text mining features and software metrics is described. More specifically, we initially provide a description of the vulnerability dataset that we utilized for the purposes of the present work. Then, we describe the generated VPMs, both software metrics-based and text mining-based ones, as well as some models that combine these two features.

### 4.1. Dataset

For the purposes of training and evaluating our models, we utilized a dataset provided by Ferenc et al. [12] that consists of multiple source code files written in JavaScript programming language retrieved from real-world open-source software projects that are available on the GitHub repository. As already mentioned, this dataset was utilized in [12] in order to build software metrics-based vulnerability prediction models. The authors of [12] collected vulnerabilities from two publicly available vulnerability databases, the Node Security Platform (NSP) (https://github.com/nodesecurity/nsp, accessed on 2 December 2021) and the Snyk Vulnerability Database (https://security.snyk.io/, accessed on 5 January 2022). Both projects try to look for insecure third-party module usages in programs. They provide command-line and web-based interfaces that can scan any Node.js module for external dependencies that are known to be vulnerable. To do so, they use a list of known vulnerabilities to search for security flaws in the version of an external module that the programs rely on.

Through this process, a list of files, which contain vulnerabilities, was obtained. For each file with vulnerabilities, they kept their GitHub Uniform Resource Locator (URL) and by traversing these URLs, they derived a set of fixing commits. Using these commits, they gathered all the code changes into a single patch file that comprised all the fixes from the repairing commits. They obtained these data with the help of the GitHub API (https://docs.github.com/en/rest, accessed on 10 March 2022). Furthermore, they recognized the parent commit of the first commit in time associated with each system’s vulnerability fix. All the functions of parent commit that were affected by the fixing modifications were considered as vulnerable, whereas the functions that were not included in the code changes were considered as non-vulnerable.

Then they employed two static code analyzers, namely escomplex (https://github.com/escomplex/, accessed on 10 March 2022) and OpenStaticAnalyzer (https://github.com/sed-inf-u-szeged/OpenStaticAnalyzer, accessed on 10 March 2022) in order to generate static software metrics. The list of the produced metrics can be seen in Table 1.

The provided dataset (http://www.inf.u-szeged.hu/ferenc/papers/JSVulnerabilityDataSet/, accessed on 5 November 2021) is structured in the format of a Comma-Separated Values (CSV) file, where each line corresponds to a JavaScript function. The columns contain information about the function name, its full path, the GitHub URL of the file where it is included and there are also 35 columns with the values of the aforementioned software metrics. There is also one last column, which is the vulnerability class (equal to one for vulnerable methods, equal to zero for non-vulnerable ones).

**Figure 3:**
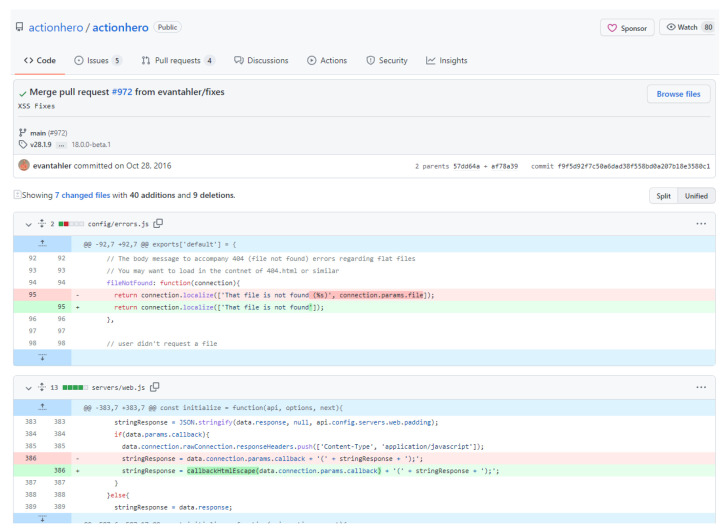
The diff file of the Cross-Site Scripting fixing commit of actionhero’s config/errors.js and servers/web.js.

In order to validate the contents of the dataset, we randomly chose and examined several samples of the dataset manually. Our evaluation procedure was based on the search of the packages of the dataset samples in the Snyk Vulnerability Database using its web-based interface. We provide an example based on the “actionhero” package, in order to allow the reader understand the procedure that we followed for verifying manually the correctness of samples of the dataset. The file “initFileServer.js”, provided by the “actionhero”, was searched in the Snyk’s web interface with the keyword “actionhero”. The interface returned two vulnerabilities, one Cross-Site Scripting (XSS) (CWE-79 (https://cwe.mitre.org/data/definitions/79.html, accessed on 10 March 2022)) and one Directory Traversal (CWE-22 (https://cwe.mitre.org/data/definitions/22.html, accessed on 10 March 2022). Subsequently, we searched the XSS vulnerability on “actionhero” and we navigated to the GitHub commit that is provided as a reference (https://security.snyk.io/vuln/npm:actionhero:20161027, https://github.com/actionhero/actionhero/commit/f9f5d92f7c50a6dad38f558bd0a207b18e3580c1, accessed on 15 March 2022). We notice that e.g., the dataset’s functions “servers/web.js” and “config/errors.js” are included in the list of changed files in the vulnerability–fixing commit, as can be seen in Figure 3.

For the purposes of the present analysis apart from the computed software metrics, we also need the actual source code of the functions, in order to extract text features that are necessary for building the text mining-based models. Although the dataset contains the GitHub URLs of the source code files and the names of the analyzed functions along with their extracted metrics, the actual source code was not readily available. To this end, we processed this CSV file and making use of the GitHub URL of each file we fetched the corresponding source code from GitHub. Utilizing the information about the start and end lines of every method, we managed to detach the source code of the methods.

**Figure 4:**
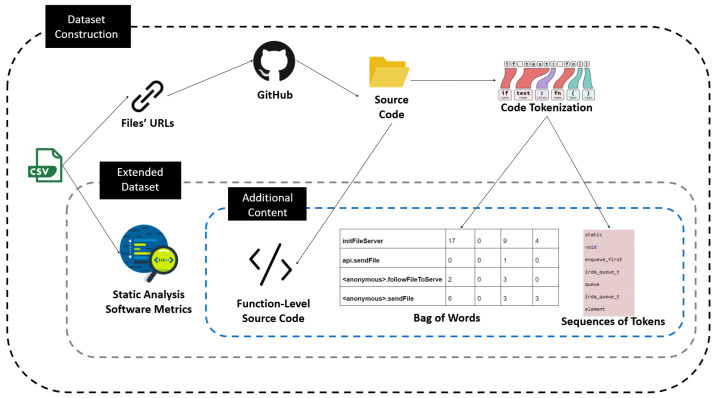
The process of constructing the overall dataset of the proposed approaches.

The overall process that we followed for extending the original dataset provided by Ferenc et al. [12] (i.e., for fetching the actual source code and extracting new text features) is illustrated in Figure 4. As can be seen by Figure 4, after downloading the dataset in CSV format provided by Ferenc et al. [12], the first step was to gather all the URLs of the function components. Then we fetched the JavaScript files’ source code from GitHub using these URLs. Subsequently, from each file we cut off the code of the functions included utilizing the start and end lines that are contained in the CSV. Every function was tokenized to construct a list of tokens per method (i.e., function). We employed two text mining techniques to extract text features, namely (i) the Bag of Words, and (ii) the Sequences of tokens. Hence, we came up with a repository of all methods’ source code, a CSV file containing the software metrics that Ferenc et al. [12] extracted, the sequences of tokens of each method and the BoW format of each method. It should be noted that all the comments were removed and also all the numbers and the strings were replaced by two unique identifiers, <numId$> and <strId$> respectively, in order to increase the generalizability of type-specific tokens [23,24]. The method’s code, along with the rest of the dataset columns of the CSV, constitute our updated dataset, which consists of 12,106 JavaScript functions, from which 1493 are vulnerable.

The final extended vulnerability dataset that contains the actual source code of the analyzed functions, their software metrics, and their text mining-based features (i.e., BoW and sequences of tokens), is made publicly available on the website with the supporting material of the present work (https://sites.google.com/view/vulnerability-prediction-data/home, accessed on 10 March 2022), along with the scripts that were utilized for extending the dataset (i.e., for fetching the actual source code and extracting the text mining-features). This will enable the replication and additional evaluation of our work, while it is also expected to facilitate future research endeavors, as researchers interested in the field of vulnerability prediction could use the dataset for building other software metric-based and text mining-based models, or further extend the dataset by extracting new features from the source code.

### 4.2. Model Construction

#### 4.2.1. Software Metrics-Based Models

As a first step in our analysis, we tried to replicate the analysis conducted by Ferenc et al. [12]. This would allow us to ensure that we are comparing against reliable results and will also allow us to utilize the dataset correctly. For this purpose, we used the dataset described in the Section 4.1, utilizing only the software metrics that were previously computed by Ferenc et al. [12] and not the textual features extracted by us. We utilized scikit-learn (https://scikit-learn.org/stable/accessed on 10 March 2022) and TensorFlow (https://www.tensorflow.org/, accessed on 10 March 2022) in order to develop ML models in Python. We trained the Decision Trees, Random Forest, Naïve Bayes, Support Vector Machine, K-Nearest Neighbors, and Deep Neural Network models because as a first step we tried to replicate the analysis conducted by Ferenc et al. [12]. Therefore, we chose the same algorithms with Ferenc et al. [12] in order to be able to compare our results directly with theirs. The descriptions of these models are listed below.

- Decision Trees: A decision tree is a decision-making algorithm that employs a tree-like model of decisions and their potential consequences, such as chance event outcomes, resource costs, and utility. It is one approach to show an algorithm made up entirely of conditional control statements.
- Random Forest (RF): Random Forest is a classification algorithm that is built from several decision trees. The new instance (i.e., input vector) is provided as input to each one of the decision trees, which predict its class. The Random Forest then gathers all of the predictions generated by each of the decision trees that belong to the Random Forest and offers a final classification. We used a 100-tree Random Forest for our studies.
- Naïve Bayes: A probabilistic classifier, the Naive Bayes classification technique is used. It is based on probability models with strong independence assumptions built in. In most cases, independence assumptions have no effect on reality. As a result, they are characterized as naive.
- Support Vector Machine (SVM): SVM is a classifier that attempts to find the best N-dimensional hyperplane (i.e., support vectors) for maximizing the margin between data points and therefore distinguishing them. To accomplish this, it aims to learn a nonlinear function by linearly mapping data points into a high-dimensional feature space.
- K-Nearest Neighbors (KNN): The outcome of k-NN classification is a class membership. An object is categorized by a majority vote of its neighbors, with the object allocated to the most common class among its k closest neighbors. If k = 1, the object is simply assigned to that single nearest neighbor’s class.
- Deep Neural Network (i.e., Multi-Layer Perceptron): A multilayer perceptron (MLP) is a type of feed-forward artificial neural network (ANN) that has multiple layers of perceptrons. MLPs are frequently used in deep learning, particularly in the construction of Deep Neural Networks (DNNs), which are ANNs with a large number of hidden layers between the input and output layers. The values of some specific variables called hyper-parameters affect the entire training process of an ANN, and hence of a DNN.

Hyper-parameter tuning was performed to determine the best hyper-parameters values for the construction of each model. We employed the Grid-search approach [25], which is often used to determine the best hyper-parameters for a model by conducting an exhaustive search through a set of hyper-parameter values for every estimator.

As already stated in Section 4.1, the dataset contains 1493 vulnerable functions in more than 12,000 functions. Hence, it is a highly imbalanced dataset, and this fact could be a barrier for the prediction task. To eliminate the risk of bias to the majority class, we examined sampling approaches to make the training set balanced. It is worth noting that sampling is only used on the training set, because re-sampling on test data introduces bias into the results. We repeated the training and the evaluation of our models implementing over-sampling until the percentage of the minority class instances was equal to the 50% of the majority class samples (similarly with Ferenc et al. [12]). We also performed under-sampling until the percentage of the samples of the majority class was equal with the ones of the 50% of the minority class. The over/under sampling technique that we utilized is the random sampling algorithm provided by imblearn (https://imbalanced-learn.org/stable/, accessed on 10 March 2022) library. Random re-sampling provides a naive technique for re-balancing the class distribution for an imbalanced dataset. It is a simple duplication (in case of over-sampling) or removal (in case of under-sampling) of some of the dataset’s samples.

The choice of independent input variables (i.e., features) is often crucial in the development of ML algorithms. Each extra feature adds a new dimension to the model, making it more complex. The “curse of dimensionality” [26], a phenomenon in which the model’s efficiency suffers as the number of input variables grows, can be triggered by a large number of input variables. Feature selection is a powerful tool for dealing with the curse of dimensionality, as it minimizes both the computational cost of modeling and the time it takes to train. In many circumstances, feature selection can even increase the model’s efficacy, as irrelevant features can have a negative impact on the model’s performance.

We used a method called Point-BiSerial Correlation (PBSC) [27,28] to investigate the statistical significance of each function-level software metric over the occurrence of vulnerabilities. PBSC can compute the correlation between a continuous and a dichotomous (i.e., binary) variable, which in this case is the existence of vulnerabilities. As we had to compare numerical variables (i.e., software metrics) with a dichotomous dependent variable (i.e., existence of vulnerabilities), we could not utilize Spearman or Pearson correlation coefficients that measure the strength of the linear relationship between variables with numerical values. We applied the PBSC method on the feature set of our dataset, and then we ranked the 35 features described in Section 4.1, in accordance with their correlation. Subsequently we filtered out the features that had a *p*-value greater than 0.05, as they do not have a statistically significant correlation within the 95% confidence interval [28]. Only six out of the 35 software metrics of the dataset were not observed to have a statistically significant correlation with the class attribute, and therefore they have been eliminated from the produced models. These six software metrics are Clone Instances, Lines of Duplicated Code, Comment Density, Documentation Lines of Code, Halstead Effort, and Halstead Time. It should be noted that during the training procedure, we gradually evaluated our models with fewer features without succeeding any improvement in the evaluation metrics, so we decided to use all the 29 features approved by the PBSC method.

#### 4.2.2. Text Mining-Based Models

In this section, we present a text mining-based approach. For this purpose, we used the dataset described in the Section 4.1, utilizing only the source code retrieved by GitHub and not the software metrics. We developed ML and DL models following two approaches:

- Bag of Words (BoW);
- Sequences of text tokens.

##### Bag of Words

In the BoW approach, a set of all the words found in the source code are considered as features used by our predictors. Each evaluated software function is represented by a list of code tokens and their associated number of occurrences in the source code. Furthermore, prediction is performed via ML models. We applied the Random Forest (RF) algorithm, which appears to be the most suitable one based on the bibliography [10,11,29], and also a DL method called Multi-Layer Perceptron (MLP) for reasons of completeness. In our BoW approach, the features that will constitute the input of the RF and MLP models are the tokens (i.e., words) that appear in the source code. More specifically, firstly, we create the vocabulary of our analysis, which actually is a list of all the tokens found in our dataset. Subsequently, we assign to each function of the dataset the number of occurrences of each token in the specific function. Hence, a table is formatted, having as lines the functions and as columns the vocabulary list. Every token that does not appear in a function gets the zero value for the specific function. A subset of a BoW dataset can be seen in Table 2. The columns of Table 2 represent some tokens of our vocabulary, while lines of Table 2 represent the name of the files in the dataset. For instance, the file initFileServer contains seventeen instances of the token ‘null’, zero instances of the token ‘this’, nine instances of the token ‘function’, and four instances of the token ‘push’.

The dataset consists of 12,942 unique tokens (i.e, a vocabulary of 12,942 tokens). The average occurrence of a token is about 1023 times. The most common term is ‘a’, with 1,159,023 occurrences, while there are several terms, such as ‘userConfig’ and ‘invalidJson’, which appear only once.

##### Sequences of Text Tokens

In the approach of sequential text tokens, each software function represents a token sequence. Each sequence includes the token in the order they appear in the source code. We feed the DL model with these sequences of tokens representing each token with a vector, which is called embedding. These embedding vector representations can be generated by several ways (see Section 2.1). In this case, the dataset’s sequences serve as the corpus for the training of the embedding vectors. We examined two sophisticated algorithms, namely word2vec and fastText, which are capable of capturing syntactic and semantic relationships between code tokens and placing these tokens in the vector space by considering their syntactic and semantic similarity. After training these embedding vectors for the vocabulary words, they can be saved for future usage, which saves time throughout the training process.

We utilized word embeddings because they convert words to vectors in a manner that vectors that are in close proximity in the vector space, correspond to words that are in close proximity in the actual source code. We also combined them with DL algorithms because they are well suited with the NLP tasks and are capable of learning sequences [24,30].

**Figure 5:**
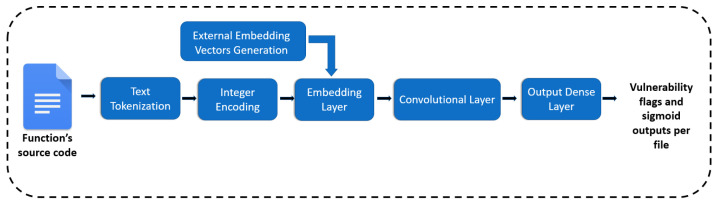
The overview of the sequences of text tokens approach.

For each dataset’s function, we define a sequence of tokens and then these tokens correspond to a unique integer. Each integer is transformed to an embedding vector using a sophisticated algorithm such as word2vec. Hence, the dataset is transformed to a list of sequences of embeddings and these embeddings serve as the numerical input to the ML model. The embedding vectors are fed into the Embedding Layer of the neural network (CNN) and finally the output layer classifies the functions as vulnerable or not, providing also the sigmoid output that indicates the confidence of the model for every prediction. An overview of the whole process is illustrated in Figure 5.

As regards to the designing of the model, a DL model was preferred and specifically the Convolutional Neural Network (CNN) that according with the experiments in [30] proved to be the most efficient and the least time intensive among the DL algorithms that can manage sequential data (i.e., LSTMs, GRUs, BiLSTMs). The CNN’s hyper-parameters were selected through extensive tuning using the Grid-search method [25] and can be found in Table 3.

### 4.3. Combination of Software Metrics and Text Mining-Based Models

As already said, one interesting research question to examine is whether the combination of software metrics and text features can lead to vulnerability prediction models with better predictive performance compared to models that focus solely on software metrics or text features (RQ2). To this end, in this section, we present the methodology of combining software metrics and text features in order to predict vulnerable software components. We attempted to design combined models by four different ways:

1. Combine software metrics with BoW features. In this approach, the occurrences of each token in any function are considered as additional features to the code metrics of the corresponding function.
2. Combine software metrics with token sequences. For this purpose, we utilized the Keras functional API (https://keras.io/guides/functional_api/, accessed on 10 February 2022), which allows us to combine different kinds of input in different layers.
3. Apply a Majority Voting approach. For each instance, the output of the text mining-based model (either BoW or sequences) was compared with the output of the software metrics-based model, and the output with the biggest probability was qualified.
4. Apply a Stacking ensemble method. The predicted probabilities of both the software metrics-based models and the two text mining-based models were used as input for another estimator called meta-classifier that is described in the Section 5.2.2.

## 5. Results & Discussion

The results of our analysis and the results of the experiments are presented in this section. All the experiments with neural networks were carried out on an NVIDIA GeForce GTX 1660 GPU running on the CUDA platform (https://developer.nvidia.com/cuda-toolkit, accessed on 10 Janury 2022). For the ML models training, we used an i5-9600K CPU at 3.70 GHz with 16 GB RAM. For the evaluation of the models, 10-fold Cross-Validation (CV) was performed. During a 10-fold CV, the overall training dataset is divided into 10 parts, from which the 9 constitute the training set and the left one constitutes the validation test. At the end of each training process, we evaluated our models based on the prediction on the validation set. In VP, the most important goal is to identify as many vulnerable software components as possible, so the Recall should be as high as possible. On the other hand, it is essential to reduce the number of FP and consequently to increase the Precision in order to make the model usable in practice. The F_2_-score is a weighted average of Precision and Recall, with Recall being more important than Precision. Hence, we have put particular focus on the F_2_-score. F_2_-score is equal to:

(1) $$F_{2}=\frac{5\times precision\times recall}{4\times precision+recall}$$

### 5.1. Comparison between Text Mining-Based and Software Metrics-Based Vulnerability Prediction Models

In this section, we focus on the first Research Question (RQ1) and we compare the utilization of software metrics and text features in Vulnerability Prediction. We present the results of our analysis and we discuss the results of each approach.

#### 5.1.1. Software Metrics Evaluation

As already stated, the first step of our experimental analysis is the replication of the work provided by Ferenc et al. [12]. Table 4 reports the evaluation results of the ML models that were built based on the software metrics that Ferenc et al. [12] statically extracted from the source code. This table sums up the results of six different algorithms regarding their accuracy, precision, recall, F_1_-score and F_2_-score with the latest to be the most critical for the vulnerability prediction case.

Table 5 presents the results produced by Ferenc et al. [12] as regarding the F_1_-score.

By comparing the results reported in Table 4 and Table 5, one can identify the convergence in the F_1_-score between our analysis and Ferenc et al. evaluation. In both cases, F-measures of Decision Trees are 72%, SVMs are close to 70%, both Naïve Bayes scores are 15%, and although Neural Network, KNN, and RF approaches have slight differences, they are close enough and most importantly, the best model produced F_1_-score close is equal to 77.62% in our case and 76% in their case. Hence, we can conclude that the software metrics-based approach using different ML models catches maximum value close to 78% with this dataset. It should be noted that none of the over/under sampling techniques that we attempted managed to provide any benefit.

The above analysis indicates that the adoption of software metrics may be a promising solution for conducting vulnerability prediction, as in all cases the F_2_-score was found to be above 70%. In our analysis, it seems that the Random Forest with 100 trees is the best approach, as apart from the relatively high F_1_ and F_2_ scores it also demonstrates high Precision (above 90%). This indicates that the model treats the problem of the many False Positives sufficiently, dealing with a well-known problem in the literature that hinders the practicality of the produced models. More specifically, low values of precision indicate that the model produces a large number of False Positives, which means that the developer would have to focus on components (e.g., functions) that are marked as vulnerable by the model but are in fact clean. In addition to this, the developer would also have to triage a large number of actually clean functions in order to spot a vulnerable one. This obviously leads to a waste of valuable resources, expressed in terms of time and effort required to spend in order to spot a vulnerability.

#### 5.1.2. Text Mining Evaluation

Subsequently, we trained and evaluated through 10-fold CV our proposed text mining based models. As regard the BoW method, the results both the prevailing Random Forest (RF) with 100 trees and the Multi-Layer Perceptron (MLP) models are reported in the Table 6:

From Table 4 and Table 6, it is clear that text mining is very beneficial to the VP task. Best BoW model succeeds almost 8% and 10% higher F_1_-score and F_2_-score respectively, in contrast with the software metrics approach, which constitutes a significant improvement. Moreover, the RF model seems to be a slightly better option than the MLP, since it overcomes MLP in both F_1_ and F_2_ scores. This is in line with the majority of the research work in the field of vulnerability prediction utilizing BoW, in which also Random Forest was found to be the best model [10,29].

In Table 7, the evaluation metrics of the sequences of tokens-based models are presented:

Based on Table 7, we could argue that the employment of DL to predict vulnerabilities, specifically using Convolutional Neural Networks (CNN), can constitute a promising method. We examined two different embedding methods, namely word2vec and FastText (https://radimrehurek.com/gensim/models/fasttext.html, accessed on 10 March 2022) algorithms. The results obtained show that the model built utilizing embedding vectors trained with word2vec are better in vulnerability prediction with respect to their F_1_-score and F_2_-score, compared to the model built utilizing embedding vectors that were trained with the FastText algorithm.

In comparison with the software metrics approach, it can be seen that the sequence-based CNN models outperform the software metrics-based models. In particular, the best CNN model (as can be seen by Table 7) achieves an F_1_-score of 85.73% and an F_2_-score of 85.62%, which is 8% and 14% higher than the F_1_-score and F_2_-score respectively of the best software metrics-based model reported in Table 4. In comparison with the BoW approach (see Table 6), the sequence-based models still demonstrate better predictive performance; however, the difference in the performance is much smaller compared to the metrics-based models, at least with respect to their F_1_-score and F_2_-score. This could be expected by the fact that those approaches are similar in nature (i.e., they are both text mining approaches), and their difference lies in the way how the text tokens are represented. In fact, the improvement that the sequence-based models introduce is that instead of taking as input the occurrences of the tokens in the code, they take as input their sequence inside the source code, potentially allowing them to detect more complex code patterns, and, thus, this improvement in the predictive performance could be attributed to those complex patterns. In general, from the above analysis one can notice that text mining-based models (either based on BoW or on the sequences of tokens) provide better results in vulnerability prediction than the software metrics-based models.

In answering to the RQ1, both text mining-based and software metrics-based models demonstrate sufficient performance in predicting the existence of vulnerabilities in software functions. However, text mining-based models outperform software metrics-based models in vulnerability prediction.

To gather it altogether, we present the Table 8 and Table 9. The Table 8 contains the different characteristics of the text mining–based and the software metrics–based models. The Table 9 includes the results of the two approaches altogether. Both tables refer to the best models of each method, based on our experiments.

**Figure 6:**
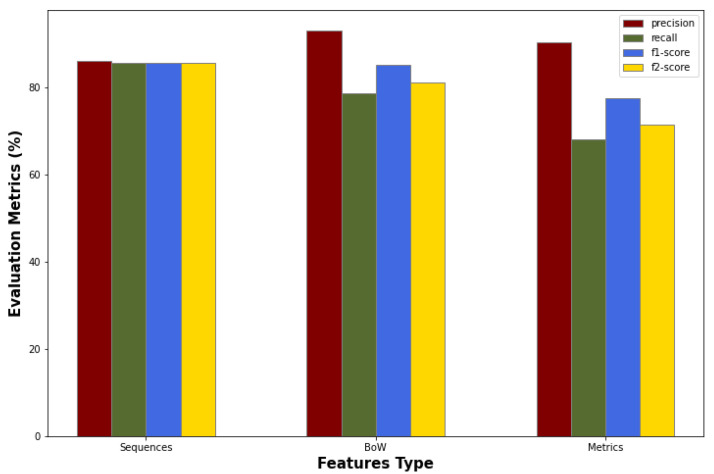
Bar chart with the evaluation metrics of the sequences of tokens, BoW and software metrics approaches.

In the Figure 6, the results of the token sequences, Bag of Words, and software metrics approaches are illustrated in the format of bar charts. In the Figure 6, it seems that the Software Metrics approach, though it turned out to be the less reliable one, it demonstrates very high precision. This implies that the model adequately addresses the problem of many False Positives, a well-known issue in the literature that impedes the practicality of the developed models. However, this may actually be due to the fact that the model favors reducing False Positives over False Negatives. This way the number of False Negatives increases, while the most important goal in the Vulnerability Prediction is to reduce the False Negatives and identify as many True Positives as possible.

### 5.2. Combination of Text Mining and Software Metrics in Vulnerability Prediction

In this section we focus on the second Research Question (RQ2) and we examine whether the combination of software metrics and text features in a unified model could lead to better predictive performance compared to the individual models focusing on a certain type of features that we have examined so far. A positive answer to this question would indicate that existing text mining-based vulnerability prediction models could benefit from the complementary utilization of selected software metrics. As already stated, we follow two broader approaches: (i) we attempt to combine text features and software metrics into a unified model, and (ii) we attempt to combine individual text mining-based and software metrics-based models through ensemble learning.

#### 5.2.1. Combining Text Mining Features and Software Metrics into a Unified Model

**Figure 7:**
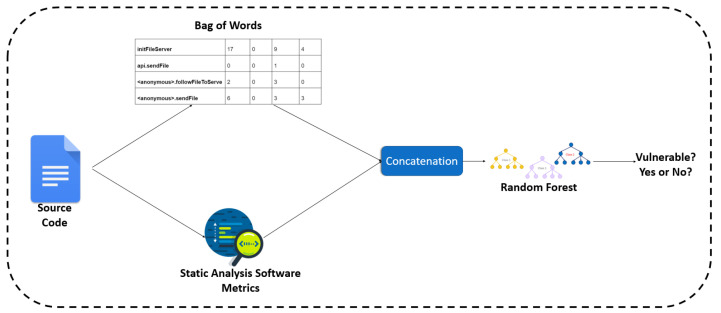
The overview of the approach combining BoW and software metrics.

In this section, we attempt to combine the two aforementioned vulnerability indicators (i.e., code metrics and text features) into a unified model. Firstly, we combined the software metrics and the text mining technique called BoW, in order to build a model that combines both types of features to generate its decision. This process requires a simple concatenation of the software metrics with the BoW’s text tokens for each function of the dataset, and utilization of the concatenated set of features to build the model. We used the RF algorithm as predictor for the combined model, as it proved to be the most trusted one for each one of the individual approaches. An overview of this approach can be found in Figure 7.

**Figure 8:**
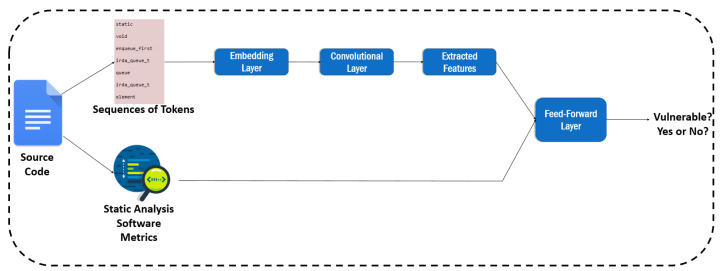
The overview of the approach combining sequences of tokens and software metrics.

Subsequently, we attempted to combine the software metrics with our second text mining technique that uses sequences of tokens. For this purpose, we utilized the Keras Functional API (https://keras.io/guides/functional_api/, accessed on 10 March 2022), which provides the capability of designing models with different inputs and outputs. Using this API, we managed to use a CNN layer along with an embedding layer in order to extract features from the sequences of tokens, then to concatenate the extracted features with the software metrics, and finally to add one feed-forward layer to receive the concatenated set of features. An overview of this method is illustrated in Figure 8. Table 10 reports the related results.

As can be seen in Table 10, no improvement in the predictive performance (compared to the performance of the best model presented in Section 5.1.2) is observed from the combination of these features. Actually, in the case of software metrics and token sequences combined, the performance is very poor, and this is why we resorted to the approach of ensemble learning (see Section 2.2).

#### 5.2.2. Combining Different Models with Ensemble Learning

**Figure 9:**
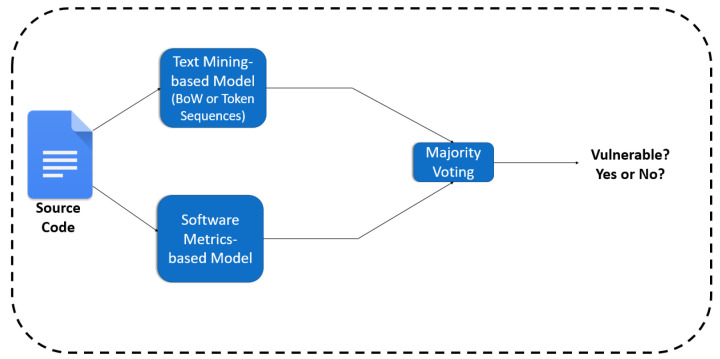
The overview of the voting approach between text mining and software metrics.

As already stated in Section 4.3, we also applied two ensemble learning techniques, namely the voting and the stacking. By employing ensemble classifiers, we aim to reduce the error of the individual classifiers by counterbalancing their predictions. As regards the voting, we adopted the soft voting (https://machinelearningmastery.com/voting-ensembles-with-python/, accessed on 10 March 2022) technique. In a soft voting ensemble, the predicted probabilities for class labels are added up and the class label with the highest sum probability is predicted. Hence, for each function, from the two applied models’ (i.e., text mining and software metrics-based) predictions the one with the higher probability is qualified (see Figure 9). Table 11 summarizes the outcome of this approach.

However, similarly to the previous experiment, voting does not improve the evaluation metrics. It seems that, in this specific dataset, the software metrics-based classifier cannot identify a relevant number of vulnerabilities which are not specified by the text mining model. We reached the same conclusion after applying the stacking classifier.

**Figure 10:**
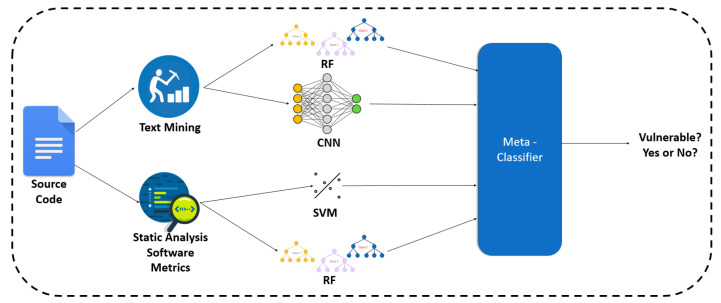
The overview of the stacking approach between text mining and software metrics.

We repeatedly trained four classifiers in nine folds of the dataset, two of them are based on software metrics (SVM, RF), and two are based on text mining (i.e., BoW, sequences of tokens). Then we made predictions with each classifier, and we saved the predicted probabilities. These probabilities constituted the input of the meta-classifier. We selected RF as a meta-classifier algorithm, based on experiments. This meta-classifier was trained on the output of the first ones, and it was evaluated in a second CV loop. Figure 10 illustrates the overview of this approach, while Table 12 presents the produced results.

Although this approach provided better results compared to the combination of features and the voting that are presented in Table 10 and Table 11 respectively, it still reaches 2% lower F_2_-score than the higher F_2_-score reached when using text-mining based CNN with word2vec embeddings (i.e., 85.62%). In simple words, the combination of statically extracted code metrics and text features (either BoW or sequences of tokens) did not manage to surpass the text mining approach, at least on this specific dataset. The fact that the ensemble learning classifiers did not produce better results leads to the conclusion that almost all the right predictions of the software metrics-based models are included in the right decisions of the text mining-based model and so, there are no errors to be compensated.

In answering the RQ2, the combination of software metrics and text features led to vulnerability prediction models with sufficient predictive performance. However, the produced models did not provide better results than the models that are based solely on text features. This suggests that, at least for the given dataset, text mining-based models, and especially those built using word embedding vectors, constitute the most accurate approach, compared to software metrics-based models and models that combine software metrics and text features.

## 6. Limitations and Threats to Validity

In this section, we discuss the limitations and validity threats of this empirical study. We discuss about both internal and external validity, but also about construct and reliability threats. The ability to generalize results is known as external validity. Because the applicability of ML models to predict vulnerabilities is investigated on a specific JavaScript dataset, the study’s findings are sensitive to external validity risks. It is always likely that a different collection of data will lead to different results. However, the chosen approaches are language agnostic, allowing developers to use them in other datasets of different programming languages. Another threat to external validity is the validity of the utilized dataset, as we cannot be sure about the validity of the labeling of the source code functions as vulnerable or not. To eliminate this risk, we performed a manual investigation on randomly selected data samples, as described thoroughly in Section 4.1. Concerning the internal validity, which refers to a study’s ability to accurately measure a causal effect in the context under investigation, the hyper-parameters chosen for the models could be biased by the dataset that was used for training. In order to address this risk, techniques for avoiding over-fitting, such as Early Stopping, were used. We also performed our evaluation process based on the Cross-Validation method in order to avoid the bias on a specific test subset.

As regards the construct validity of the experimented prediction models, we employed the scikit-learn library’s implementation of machine learning techniques, which is widely regarded as a dependable tool. For the purposes of the DL models, we utilized the provided by TensorFlow Keras framework, which is a highly reliable and very popular framework.

Finally, the prospect of replicating this study poses a risk of reliability. We give an experimental package containing both the dataset and the scripts that were utilized for our analysis and the vulnerability prediction models creation to make replication studies easier. This information can be accessed online (https://sites.google.com/view/vulnerability-prediction-data/home, accessed on 10 March 2022).

## 7. Conclusions

In the present paper, we evaluated the predictive performance of text mining-based and software metric-based vulnerability prediction models. We also examined whether the combination of software metrics and text features could lead to better vulnerability prediction models, as opposed to models built solely on text mining features or software metrics. More specifically, for the purposes of the present study, we utilized and extended a vulnerability dataset constructed by Ferenc et al. [12], labeled with vulnerabilities in function level, in order to investigate mainly, whether the adoption of text mining surpasses the software metrics approach (adopted by Ferenc et al. [12]) and subsequently, whether the combination of these kinds of features could be proved beneficial. We evaluated our approach using 10-fold cross validation focusing chiefly on the F_2_-score. Our analysis led to the conclusion that text mining is an effective solution for vulnerability prediction, while it is superior to software metrics utilization. More specifically, both Bag of Words and token sequences approaches provided better results than the software metrics-based models. Another interesting observation that was made by our analysis is that the combination of software metrics with text features did not lead to more accurate vulnerability prediction models. Although their predictive performance was found to be sufficient, it did not manage to surpass the predictive performance of the already strong text mining-based vulnerability prediction models. In particular, neither the simple concatenation nor the more sophisticated ensemble learning techniques (i.e., voting, stacking) managed to surpass the text mining-based models, and especially those built using sequences of word embedding vectors.

Several directions for future work can be identified. Firstly, since there is always the threat of generalizability, the present analysis needs to be repeated in the future, utilizing different datasets preferably of different programming languages, in order to investigate whether this observation is general or holds only for a specific language or dataset. Different DL architectures may also prove to be beneficial to our attempt to capture patterns in the source code that are indicative of vulnerability existence. In the present paper, we utilized popular word embedding algorithms (i.e., word2vec and fastText) for the representation of the text tokens. However, different embedding architectures could provide better results. For this purpose, it would be an interesting topic to examine transformer-based pre-trained models such as BERT and codeBERT [31]. Additional software metrics or textual features could be also examined.

As regards the broader Vulnerability Prediction field, there are many open challenges that we could examine. It is critical to increase the performance of existing VPMs so they can predict vulnerability hotspots in software projects they have never seen before. It is important to study the ability of recent approaches based on abstract syntax trees [32,33] to achieve high-accurate cross-project performance. Furthermore, previously underexplored software-related characteristics could be evaluated for their relevance to software security and their ability to detect software flaws. The newly discovered characteristics could be used to improve vulnerability prediction models’ predictive performance. Another very interesting open challenge is also the explainability of the behavior of the ML models and the investigation of which features and tokens affect more the decision making of the models. We could examine whether the models generalize or they learn something specific to the training dataset, with a view to the cross-project vulnerability prediction.

## Figures and Tables

**Table 1:** The statically extracted software metrics.

Metric	Description
CC	Clone Coverage
CCL	Clone Classes
CCO	Clone Complexity
CI	Clone Instances
CLC	Clone Line Coverage
LDC	Lines of Duplicated Code
McCC, CYCL	Cyclomatic Complexity
NL	Nesting Level
NLE	Nesting Level without else-if
CD, TCD	(Total) Comment Density
CLOC, TCLOC	(Total) Comments Lines of Code
DLOC	Documentation Lines of Code
LLOC, TLLOC	(Total) Logical Lines of Code
LOC, TLOC	(Total) Lines of Code
NOS, TNOS	(Total) Number of Statements
NUMPAR, PARAMS	Number of Parameters
HOR_D	NR. Of Distinct Halstead Operators
HOR_T	Nr of Total Halstead Operators
HON_D	NR. Of Distinct Halstead Operands
HON_T	Nr of Total Halstead Operands
HLEN	Halstead Length
HVOC	Halstead Vocabulary Size
HDIFF	Halstead Difficulty
HVOL	Halstead Volume
HEFF	Halstead Effort
BUGS	Halstead Bugs
HTIME	Halstead Time
CYCL_DENS	Cyclomatic Density

**Table 2:** A Bag of Words (BoW) subset of the dataset.

Function Name	Null	This	Function	Push
initFileServer	17	0	9	4
api.sendFile	0	0	1	0
<anonymous>.followFileToServe	2	0	3	0
<anonymous>.sendFile	6	0	3	3

**Table 3:** The chosen Hyper-parameters of the Convolutional Neural Network (CNN) model.

Hyper-Parameter Name	Hyper-Parameter Value
Number of Layers	3 (Embedding-Convolutional-Dense)
Number of Convolutional Layers	1 (1D CNN)
Embedding Size	300
Number of Filters	128
Kernel Size	5
Pooling	Global Max Pooling
Weight Initialization Technique	Glorot Uniform (Xavier)
Learning Rate	0.01
Gradient Descent Optimizer	Adam
Batch Size	64
Activation Function	Relu
Output Activation Function	Sigmoid
Loss Function	Binary cross entropy
Maximum Epochs	100
Early Stopping Patience	10
Monitoring Metric	Recall

**Table 4:** Evaluation results of software metrics based models.

Evaluation Metric	KNN	RF	Decision Trees	SVM	Naive Bayes	ANN
Accuracy (%)	93.10	95.16	93.19	94.34	84.40	91.72
Precision (%)	72.85	90.42	73.10	94.62	23.75	73.65
Recall (%)	70.60	68.05	71.07	57.40	12.06	54.06
F_1_-score (%)	71.66	77.62	72.04	71.43	15.92	61.24
F_2_-score (%)	71.01	71.58	71.45	62.29	13.35	56.62

**Table 5:** Evaluation results of software metrics-based models according to Ferenc et al.

Evaluation Metric	KNN	RF	Decision Trees	SVM	Naive Bayes	ANN
F_1_-score (%)	76	71	72	67	15	71

**Table 6:** Evaluation results of BoW models.

Evaluation Metric	RF	MLP
Accuracy (%)	96.64	94.13
Precision (%)	93.16	77.82
Recall (%)	78.57	82.65
F_1_-score (%)	85.20	79.03
F_2_-score (%)	81.08	80.76

**Table 7:** Evaluation results of models that are based on sequences of tokens.

Evaluation Metric	CNN with Word2Vec Embeddings	CNN with FastText Embeddings
Accuracy (%)	96.48	92.94
Precision (%)	86.12	66.64
Recall (%)	85.60	88.08
F_1_-score (%)	85.73	75.66
F_2_-score (%)	85.62	82.58

**Table 8:** A table with the characteristics of both text mining–based and software metrics–based models.

	Sequences of Tokens	Bag of Words	Software Metrics
Machine/Deep Learning (ML/DL)	DL	ML	ML
Type of model	Neural network	Random Forest	Random Forest
Type of input	Embedded Sequences	Tokens occurrences	Numerical values
Particular features	Convolutional	100 trees	100 trees

**Table 9:** A table with the evaluation scores of both text mining–based and software metrics–based models.

Evaluation Metric	Sequences of Tokens	Bag of Words	Software Metrics
Accuracy (%)	96.48	96.64	95.16
Precision (%)	86.12	93.16	90.42
Recall (%)	85.60	78.57	68.05
F_1_-score (%)	85.73	85.20	77.62
F_2_-score (%)	85.62	81.08	71.58

**Table 10:** Combination of text mining and software metrics.

Evaluation Metric	Software Metrics and BoW	Software Metrics and Token Sequences
Accuracy (%)	96.32	72.88
Precision (%)	93.55	30.57
Recall (%)	75.35	68.68
F_1_-score (%)	83.43	40.84
F_2_-score (%)	78.38	52.85

**Table 11:** Voting classification between text mining and software metrics based models.

Evaluation Metric	Voting-Soft. Metrics and BoW	Voting-Soft. Metrics and Tokens
Accuracy (%)	96.23	95.93
Precision (%)	94.54	88.42
Recall (%)	73.75	77.09
F_1_-score (%)	82.81	82.32
F_2_-score (%)	77.11	79.09

**Table 12:** Stacking classifier evaluation.

Evaluation Metric	Stacking-Software Metrics and Text Mining
Accuracy (%)	96.78
Precision (%)	90.75
Recall (%)	82.31
F_1_-score (%)	86.29
F_2_-score (%)	83.86

## Data Availability

Data that support the reported results can be found at https://sites.google.com/view/vulnerability-prediction-data/home (accessed on 10 March 2022).

## References

[1] Shin Y., Williams L. Is complexity really the enemy of software security?. Proceedings of the 4th ACM Workshop on Quality of Protection.

[2] Shin Y., Williams L. An empirical model to predict security vulnerabilities using code complexity metrics. Proceedings of the Second ACM-IEEE International Symposium on Empirical Software Engineering and Measurement.

[3] Chowdhury I., Zulkernine M. (2011). Using complexity, coupling, and cohesion metrics as early indicators of vulnerabilities. J. Syst. Archit..

[4] Pang Y., Xue X., Wang H. Predicting vulnerable software components through deep neural network. Proceedings of the 2017 International Conference on Deep Learning Technologies.

[5] Li Z., Zou D., Xu S., Ou X., Jin H., Wang S., Deng Z., Zhong Y. (2018). Vuldeepecker: A deep learning-based system for vulnerability detection. arXiv.

[6] Zheng J., Williams L., Nagappan N., Snipes W., Hudepohl J.P., Vouk M.A. (2006). On the value of static analysis for fault detection in software. IEEE Trans. Softw. Eng..

[7] Gegick M., Williams L. Toward the use of automated static analysis alerts for early identification of vulnerability-and attack-prone components. Proceedings of the Second International Conference on Internet Monitoring and Protection (ICIMP 2007).

[8] Neuhaus S., Zimmermann T., Holler C., Zeller A. Predicting vulnerable software components. Proceedings of the 14th ACM Conference on Computer and Communications Security.

[9] Hovsepyan A., Scandariato R., Joosen W., Walden J. Software vulnerability prediction using text analysis techniques. Proceedings of the 4th International Workshop on Security Measurements and Metrics.

[10] Walden J., Stuckman J., Scandariato R. Predicting vulnerable components: Software metrics vs text mining. Proceedings of the 2014 IEEE 25th International Symposium on Software Reliability Engineering.

[11] Zhang Y., Lo D., Xia X., Xu B., Sun J., Li S. Combining software metrics and text features for vulnerable file prediction. Proceedings of the 2015 20th International Conference on Engineering of Complex Computer Systems (ICECCS).

[12] Ferenc R., Hegedűs P., Gyimesi P., Antal G., Bán D., Gyimóthy T. Challenging machine learning algorithms in predicting vulnerable javascript functions. Proceedings of the 2019 IEEE/ACM 7th International Workshop on Realizing Artificial Intelligence Synergies in Software Engineering (RAISE).

[13] Sagi O., Rokach L. (2018). Ensemble learning: A survey. Wiley Interdiscip. Rev. Data Min. Knowl. Discov..

[14] Subramanyam R., Krishnan M.S. (2003). Empirical analysis of ck metrics for object-oriented design complexity: Implications for software defects. IEEE Trans. Softw. Eng..

[15] Goyal P.K., Joshi G. QMOOD metric sets to assess quality of Java program. Proceedings of the 2014 International Conference on Issues and Challenges in Intelligent Computing Techniques (ICICT).

[16] Mikolov T., Chen K., Corrado G., Dean J. (2013). Efficient estimation of word representations in vector space. arXiv.

[17] Breiman L. (1996). Bagging predictors. Mach. Learn..

[18] Bauer E., Kohavi R. (1999). An empirical comparison of voting classification algorithms: Bagging, boosting, and variants. Mach. Learn..

[19] Kalouptsoglou I., Siavvas M., Tsoukalas D., Kehagias D. Cross-project vulnerability prediction based on software metrics and deep learning. Proceedings of the International Conference on Computational Science and Its Applications.

[20] Moshtari S., Sami A., Azimi M. (2013). Using complexity metrics to improve software security. Comput. Fraud. Secur..

[21] Moshtari S., Sami A. Evaluating and comparing complexity, coupling and a new proposed set of coupling metrics in cross-project vulnerability prediction. Proceedings of the 31st Annual ACM Symposium on Applied Computing.

[22] Yu Z., Theisen C., Sohn H., Williams L., Menzies T. (2018). Cost-aware vulnerability prediction: The HARMLESS approach. arXiv.

[23] Russell R., Kim L., Hamilton L., Lazovich T., Harer J., Ozdemir O., Ellingwood P., McConley M. Automated vulnerability detection in source code using deep representation learning. Proceedings of the 2018 17th IEEE International Conference on Machine Learning and Applications (ICMLA).

[24] Dam H.K., Tran T., Pham T.T.M., Ng S.W., Grundy J., Ghose A. (2018). Automatic feature learning for predicting vulnerable software components. IEEE Trans. Softw. Eng..

[25] Bergstra J., Bardenet R., Bengio Y., Kégl B. (2011). Algorithms for hyper-parameter optimization. Adv. Neural Inf. Process. Syst..

[26] Chen L. (2009). Curse of dimensionality. Encyclopedia of Database Systems.

[27] Kremenek T., Ashcraft K., Yang J., Engler D. (2004). Correlation exploitation in error ranking. Acm Sigsoft Softw. Eng. Notes.

[28] Varma S. (2006). Preliminary Item Statistics Using Point-Biserial Correlation and p-Values.

[29] Scandariato R., Walden J., Hovsepyan A., Joosen W. (2014). Predicting vulnerable software components via text mining. IEEE Trans. Softw. Eng..

[30] Kalouptsoglou I., Siavvas M., Kehagias D., Chatzigeorgiou A., Ampatzoglou A. An Empirical Evaluation of the Usefulness of Word Embedding Techniques in Deep Learning-based Vulnerability Prediction. Proceedings of the EuroCybersec2021, Lecture Notes in Communications in Computer and Information Science.

[31] Bagheri A., Hegedűs P. A Comparison of Different Source Code Representation Methods for Vulnerability Prediction in Python. Proceedings of the International Conference on the Quality of Information and Communications Technology.

[32] Bilgin Z., Ersoy M.A., Soykan E.U., Tomur E., Çomak P., Karaçay L. (2020). Vulnerability prediction from source code using machine learning. IEEE Access.

[33] Cao S., Sun X., Bo L., Wei Y., Li B. (2021). BGNN4VD: Constructing Bidirectional Graph Neural-Network for Vulnerability Detection. Inf. Softw. Technol..

